# Production of activated carbon from sawdust and its efficiency in the treatment of sewage water

**DOI:** 10.1016/j.heliyon.2021.e05960

**Published:** 2021-01-15

**Authors:** Temitayo E. Oladimeji, Babatunde O. Odunoye, Francis. B. Elehinafe, Oyinlola, R. Obanla, Olayemi, A. Odunlami

**Affiliations:** Department of Chemical Engineering, Covenant University, Ota, Ogun State, Nigeria

**Keywords:** Activated carbon, Treatment, Sewage water, Water pollution

## Abstract

When water is contaminated and rendered unfit for drinking, it is regarded as waste, which leads to water pollution. Several works have been done to control water pollution, yet this topic is still a point of concern up to date. The study involves the production of activated carbon (AC) using sawdust to treat sewage water obtained from Covenant University. The following conditions were investigated; activation time and temperature, activating agent concentration, and impregnation time. The AC was characterized by measuring ash content, iodine value, moisture, and volatile matter content. The optimum activated carbon prepared in this study had iodine of 1628.95 mg/gm, while the minimum activated carbon had an iodine of 470.41 mg/gm. According to standard procedure, the sewage water sample was characterized physio-chemically before and after treatment using activated carbon as an adsorbent. The results obtained indicated considerable improvement in the quality of the water. When optimum activated carbon was used for treatment, pH value changed from 7.7 to 7.10, biochemical oxygen demand (BOD) was reduced from 288 mg/l to 20 mg/l and Total dissolved solids (TDS) reduced from 183.7 mg/l to 16.4 mg/l, Total suspended solids (TSS) reduced 232 mg/l to 15.7 mg/l. When minimum activated carbon was used for treatment, pH value changed from 7.7 to 7.60, BOD was reduced from 288 mg/l to 112.2 mg/l, and TDS reduced from 232 mg/l to 174 mg/l, TSS reduced 183.7 mg/l to 103 mg/l. The results obtained led to the conclusion that the produced activated carbon effectively treats the above-stated water quality parameters.

## 1 Introduction

Water pollution has become a threat to the world; it has been proposed to be the highest global indicator of deaths and infections (World Pollution, 2015), and that it sums up to the deaths of over 14,000 individuals daily. An estimate of about 1,000 Indian children experience death due to diarrhea daily, and 90 % of China cities also grieve from some level of water pollution (World Pollution, 2015). A developing country like Nigeria is still struggling with pollution complications as well. Careless disposal of Industrial wastewater can render the water body contaminated, reducing the amount of dissolved oxygen and Biological oxygen demand (BOD) in the water.

Several treatment processes have been carried out to improve water quality (Ekpete and Horsfall, 2011; Tchobanoglous et al., 2003). However, these processes' shortcomings include toxic sludge formation, complicated process, high cost of maintenance, and operational cost. Therefore, a need arises to investigate better processes that will serve as an alternative to the sophisticated processes. One of which is the use of activated carbon obtained from processed agricultural products. The most frequently used raw material to produce activated carbon is coal (anthracite, lignite, and bituminous) and locally sourced vegetable origin waste such as corn cobs, palm kernel shell, coconut shell, etc. These agricultural products create a substantial quantity of waste, which can be utilized using suitable technologies to produce useful products like activated carbon (Asadullah et al., 2007; Jun et al., 2010).

Activated carbon (AC) is a broadly exploited industrial adsorbent that comprises carbonaceous material with a porous configuration and increased surface area (Asadullah et al., 2007; Yeganeh et al., 2006). Activated carbon has been documented in Sanni et al. (2017), and Adinata et al. (2007) possess several factors that include a high adsorption rate and permeable structure. Research on activated carbon shows its application in various industrial processes such as food (Bamforth, 2006), beverage (Erickson, 1995), and textile industries (Cukierman, 2013). AC is one of the commonly used adsorbents in treating wastewater and reduce the BOD and chemical oxygen demand (Ghodale and Kankal, 2014).

Activated carbon can be produced in two mediums, which are physical and chemical activation processes. The single-stage process known as chemical activation involves utilizing an activating agent before the carbonization of material. It is carried out at low temperatures to boost the porous structure formation. The physical activation method includes carbonizing the charcoal's material in oxygen and activation (Jun et al., 2010).

Sawdust is a waste byproduct of wood used in construction work and furniture. Sawdust conserves moisture, prevents weeds and grass, and keeps the plant roots cool when spread around plants and shrubs. Parks et al. (2010) found sawdust suitable for raising tomato and cucumber plants. Adebakin et al. (2012) also showed the use of sawdust as an admixture in hollow concrete production. Shukla (2002) demonstrated the role of sawdust in the removal of unwanted materials from water. Despite the advantages of sawdust, its disposal might create environmental issues; hence a need arises to solve this problem. Sawdust is low in ash and high in carbon content (50% w/w) (Yeganeh et al., 2006). As documented by Elehinafe et al. (2019), sawdust's carbon content ranges from 77.51% to 93.59%, and ash content as low as 0.08%. Soleimani and Kaghazchi (2007) gave the carbon and ash weight percentage (wt%) of some other agricultural waste products such as almond shell, bagasse, apricot stone, and walnut shell 50.30/1.54 wt%, 46/3.4 wt%, 50.50/3.2 wt%, and 49/1.7 wt%, respectively.

This study aims at measuring the efficiency of activated carbon produced from sawdust in sewage water treatment. Due to its advantage over other materials, it is used as the raw material for this study. The material's choice is the source of wood in Nigeria and the relatively low price of the raw material.

## 2 Materials and methods

### 2.1 Materials/equipment

Sawdust, Automated sieve shaker, Furnace, Oven, Incubator, Retort stand, Beakers, Glass bottles, Sewage water, Distilled water, Concentrated phosphoric acid, Anhydrous potassium phthalate, Sodium hydrogen carbonate, Potassium dichromate, potassium iodide, iodine and Silver sulfate.

### 2.2 Methods

**Figure 1:**
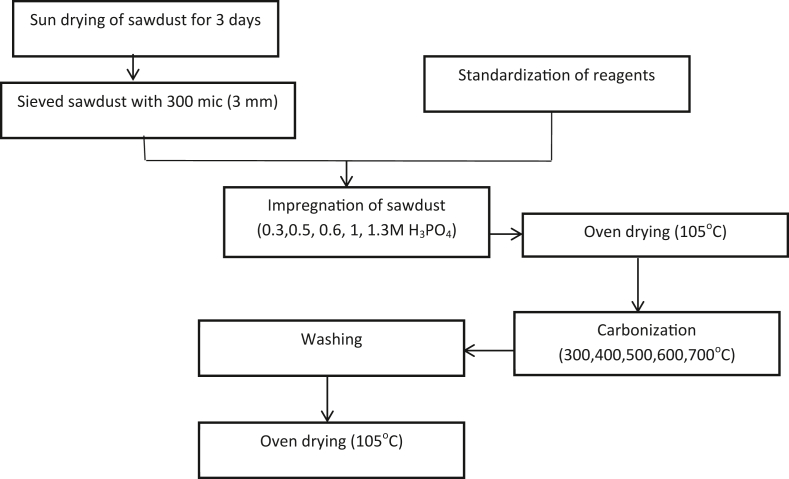
A block diagram of Production of Activated Carbon.

The processes involved in producing activated carbon and sewage wastewater treatment are presented in Figure 1 and discussed in the following sections.

#### 2.2.1 Pre-treatment of sawdust

The sawdust was collected from a sawmill in Ota, Ogun State, Nigeria. The sawdust was dried for three days and then sieved at the 300-mesh size (0.3mm) to obtain it in its powdery form, followed by washing using warm water, rinsing with distilled water, and drying at 105 °C temperature for 3 h in the oven and allowed to cool in desiccators.

#### 2.2.2 Production of activated carbon with varying carbonization temperatures

Five different dried sawdust samples were carbonized in a muffle furnace at temperatures 300 °C–700 °C for 1 h each. The carbonized samples were chemically activated using phosphoric acid of 1 mol for 24 h and then charged into the furnace at 300 °C, 400 °C, 500 °C, 600 °C, 700 °C) for 1 h, respectively. After activation, the samples were rinsed using distilled water to eliminate residual inorganic matter and excess unreacted chemical agents, which may cause unfavorable degradable reactions later. The samples' drying was done in an oven again at 105 °C till a constant weight of activated carbon samples was obtained.

#### 2.2.3 Production of activated carbon with varying concentrations of an activating agent

This result entails investigating the effectiveness of activating agent on the activated carbon, which involves determining the iodine value of the activated carbon produced.

Five different dried sawdust samples were carbonized in a muffle furnace at different temperatures 600 °C, for 1 h each. The carbonized samples were chemically activated using phosphoric acid at different concentrations of 0.3 M, 0.5 M,0.6 M,1 M, and 1.3M, each for 24 h, and then charged into the furnace at the temperatures 600 °C for 1 h. After activation, the samples were rinsed using distilled water to eliminate residual inorganic matter and excess unreacted chemical agents, which may cause unfavorable degradable reactions later. The samples' drying was done in an oven again at 105 °C till a constant weight of activated carbon samples was obtained.

#### 2.2.4 Production of activated carbon with varying impregnation time

This stage investigates impregnation time on the activated carbon, which involves determining the iodine value of the activated carbon produced.

Five different samples of the dried sawdust were carbonized in a muffle furnace at different temperatures 600 °C at different impregnation times of 10 h, 15 h, 20 h, and 24 h 30hrs. The carbonized samples were chemically activated using phosphoric acid of 1 M at 600 °C in a furnace. After activation, the samples were rinsed using distilled water to eliminate residual inorganic matter and excess unreacted chemical agents, which may cause unfavorable degradable reactions later. The samples' drying was done in an oven again at 105 °C till a constant weight of activated carbon samples was obtained.

#### 2.2.5 Production of optimum quality of activated carbon with optimum conditions

Five different samples of the dried sawdust were carbonized in a muffle furnace at optimum temperatures 600 °C for an optimum period of 30 h. The carbonized samples were chemically activated using phosphoric acid of optimum 1M at 600 °C at an optimum 75 min in a furnace. After activation, the samples were rinsed using distilled water to eliminate residual inorganic matter and excess unreacted chemical agents, which may cause unfavorable degradable reactions later. The samples' drying was done in an oven again at 105 °C till a constant weight of activated carbon samples was obtained.

#### 2.2.6 Production of minimum quality of activated carbon using minimum conditions

Five different samples of the dried sawdust were carbonized in a muffle furnace at minimum temperatures 300 °C for a minimum period of 10 h. The carbonized samples were chemically activated using phosphoric acid of a minimum 0.3 M at 300 °C at a minimum of 90 min in a furnace. After activation, the samples were rinsed using distilled water to eliminate residual inorganic matter and excess unreacted chemical agents, which may cause unfavorable degradable reactions later. The samples' drying was done in an oven again at 105 °C till a constant weight of activated carbon samples was obtained.

#### 2.2.7 Standardization of iodine solution and determination of absorptivity of adsorbent

The iodine value majorly measures the absorptive power and macroporosity of the activated carbon. 20g of potassium iodide was dissolved in 400ml of distilled water, and then 13g of iodine was added and stirred to dissolve, after which distilled water was added to make it up to 1 L. 25 ml of standardized iodine solution (SIS) was added to 0.5g of activated carbon from each stage in separate beakers, and the mixtures were filtered. After that, the 20ml filtrate was titrated with the standard thiosulphate. Finally, a blank titration was also performed, which involve the titration of 20cm^3^ of SIS not treated with activated carbon.

Therefore, iodine value (IV) was calculated using Eq. (1) by Yusuff et al. (2012);

(1) $$\text{Iodine}\;\text{Value}\;\left(\text{IV}\right)=\frac{M_{s}\left(V_{B}-V_{A}\right)}{2M_{AC}}$$

Where $M_{s}$= molarity of thiosulphate. Solution (TS).

V_B_ = volume of TS at blank titration
V_A_ = volume of TS at AC treatment
M_AC_ = Mass of AC in grams

### 2.3 Characterization of produced activated carbon

#### 2.3.1 Moisture content

1 g of the activated sample was put in the crucible and covered with a lid. The total mass of the sample with the container and lid was measured. The sample filled container was then put in an oven without the lid. The temperature of the oven was set to 110 °C for 3 hours until the weights of samples were constant. The same procedure was repeated in triplicate for the adsorbent. The Moisture content was calculated using Eq. (2).

(2) $$\text{Moisture}\;\text{content}\;\left(\text{\%}\right)=100\times \frac{\left(B-F\right)}{\left(B-G\right)}$$

where, B = mass of container with lid + original sampling.

F = mass of container with lid + dried sampling
G = mass of container with a lid.

#### 2.3.2 Ash content

10g of the sample was put in an open crucible, and the total mass was weighed. The sample was then placed in a furnace at a temperature of 900 °C for 3 h. The sample was cooled to room temperature and weighed. The same procedure was repeated in triplicate for the adsorbent. Ash content was calculated using Eq. (3):

(3) $$\text{Ash}\;\text{content}\;\left(\text{\%}\right)=100\times \frac{\left(A-C\right)}{\left(K-C\right)}$$

where, C = mass of empty crucible in g.

K = mass of crucible + original sample
A = mass of crucible + ash sample in g

#### 2.3.3 Volatile matter content

It involves the addition of 1g of sample to a crucible covered with a lid. Total mass was weighed. The sample and the covered crucible were then placed in the furnace set at 900 °C for 7 min. The sample was cooled to room temperature and weighed. The same procedure was repeated in triplicate for the adsorbent. Ash content was calculated using Eq. (4):

(4) $$\text{Volatile}\;\text{matter}\;\text{content}\;\left(\text{\%}\right)=100\times \frac{\left(100\left(B-F\right)-\text{Mc}\left(\text{B}-\text{G}\right)\right)}{\left(B-G\right)\left(100-\text{Mc}\right)}$$

where, B = mass in g of crucible, lid, and sample before heating.

F = mass in g of crucible, lid, and sample after heating.
G = mass in g of empty crucible and lid.
M_c_ = Moisture content of sample in (%)

### 2.4 Characterization of treated and untreated wastewater

#### 2.4.1 Total dissolved solids

This result was obtained by evaporating a known volume of water sample to dryness, leaving the residue. The residue was cooled to room temperature and weighed. The same procedure was repeated in triplicate to the known volume of the water sample. The residue was calculated between the differences in weight.

#### 2.4.2 Total suspended solids

This result was obtained by weighing filtrate from filtering a known volume of the water sample.

(5) Total Solids = Total dissolved solids + Total suspended solids

#### 2.4.3 Biochemical oxygen demand

Four different bottles were used to collect wastewater samples with 250 ml measurement, and 1.5 ml of Winkler's Solution was added to each bottle, and precipitates were formed. This precipitate was dissolved with 2 ml concentrated H_2_SO_4_, which later form a golden-brown solution. Also, 3 drops of the starch indicator were added to 50 ml of the solution and further titrated with Sodium thiosulphate of 0.2 M solution, which changes the color to colorless. The remaining samples were covered with black cellophane bags at 290 °C–300 °C room temperature for a few days to prevent light penetration. The procedure was repeated for five consecutive days for each of the four samples. 0.2 M volume of sodium thiosulphate used was recorded.

(6) $$C_{A}=\left[\frac{C_{B}V_{B}}{V_{A}}\right]\times \frac{n_{A}}{n_{B}}\times \frac{32g0_{2}}{mol}\times 1000\;mg/g$$

where,

C_A_ = concentration of dissolved oxygen (DO) in the polluted sample, (mg/l)
V_A_ = volume of polluted oil sample for titration (50ml)
C_B_ = Concentration of sodium thiosulphate solution. (0.2M)
V_B_ = Titre value or volume of sodium thiosulphate used for titration.

From the stoichiometric equations of the Winkler's test for dissolved oxygen].

n_A_ = number of O_2_ = 1
n_B_ = number of moles of sodium thiosulphate = 4

(7) BOD_5_ = DO_O_ - DO_5_

Where,

DO_O_ = Dissolved oxygen concentration at the beginning (zero time)
DO_5_ = Dissolved oxygen concentration after 5 days incubation period

#### 2.4.4 pH determination

2.0g of the activated carbon was weighed out using a sensitive weighing balance. The weighed activated carbon was washed thoroughly for 5 m with 30 ml distilled water and filtered using a filter paper, and the pH of the filtrate was measured using a pH meter by dipping the probe of the pH meter in the filtrate sample. This procedure was repeated in triplicate for each sample of the activated carbon.

## 3 Result and discussion

In this section, the result of experiments from the laboratory was presented and described as follows;

### 3.1 Effect of activation temperature on activated carbon with an iodine value

**Figure 2:**
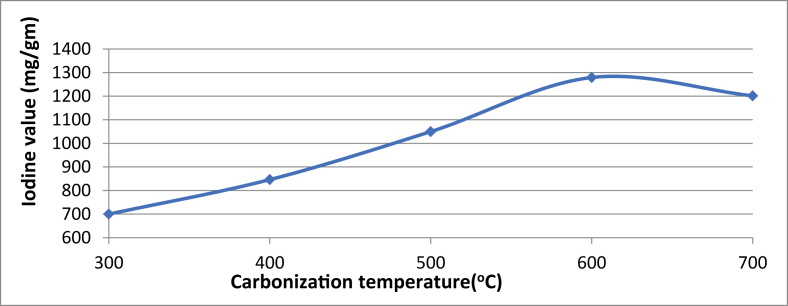
Effect of activation temperature on activated carbon with iodine value.

Temperature is a major factor in the pore arrangement of AC, which goes a long way to enhance the adsorptive ability (Hu and Vansant, 1995). The graph, Figure 2 indicates a steady increase in iodine value for carbonization temperature while it decreases at 600^o^C–700 °C; this is also reported by (Subhashree Pradhan, 2011). This decrease can be traced to excessive carbonization, resulting in distortion of pore walls, which affects the particle's micropore structure. Therefore, the optimum operating temperature used to produce AC using sawdust is 600 °C.

### 3.2 Effect of impregnation time on activated carbon with an iodine value

**Figure 3:**
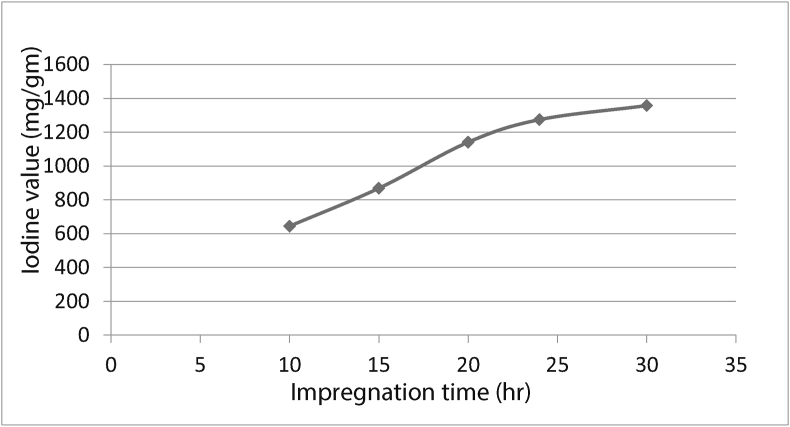
Effect of impregnation time on activated carbon with iodine value.

The iodine level increased steadily with impregnation time (IT)has presented in Figure 3. At 30 h, an equilibrium state was attained, which is the optimum IT reached.

### 3.3 Effect of carbonization time on activated carbon with an iodine value

**Figure 4:**
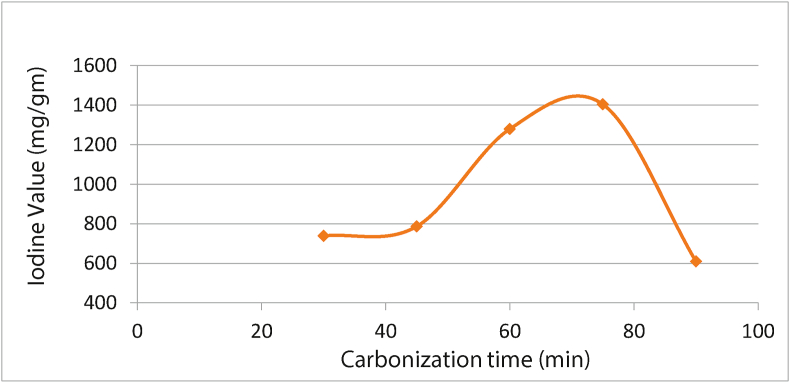
Effect of carbonization time on activated carbon with iodine value.

There were an irregular increase and decrease in the iodine value (IV) relative to its carbonization time (Figure 4); this suggests the excessive activation of the produced AC, thereby facilitating its conversion from micropores to mesopores and finally macropores.

### 3.4 Effect of concentration of phosphoric acid on activated carbon with iodine value

**Figure 5:**
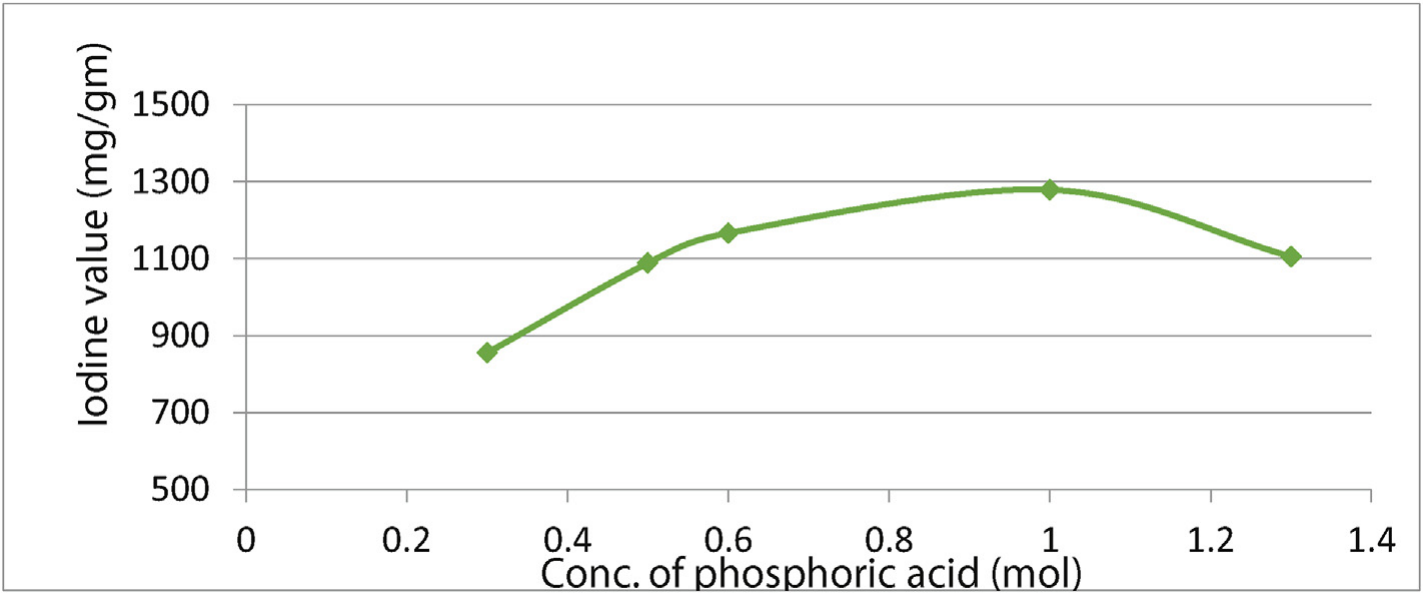
Effect of concentration of phosphoric acid on activated carbon with iodine value.

The IV increases to 1 mol of phosphoric acid (H_3_PO_4_)_,_ which means that an increase in the activating agent will increase the porosity and absorptivity performance of the AC produced (Figure 5). A decrease in the micropore structure was observed at concentrations above 1 mol H_3_PO_4_ due to excessive carbonization; hence 1 mol was chosen as the optimum temperature and 1279.05 mg/gm, optimum IV.

### 3.5 Characterization of optimum and minimum activated carbon

The optimum and minimum conditions as presented Figures 2, 3, 4, and 5 above was summarized in Table 1 the optimum rate activated carbon was derived from optimum conditions and vice versa.

**Table 1:** Optimum and minimum activated carbon.

conditions	Optimum conditions	Minimum conditions
Concentration of H_3_PO_4_	1 mol	0.3 mol
Impregnation time (IT)	30 h	10 h
Carbonization time	75 min	90 min
Carbonization temperature	600 °C	300 °C

As presented in Table 2, Moisture content signifies the amount of water present in the AC. The extent of absorptivity of the produced AC is dependent on the environment (dry or humid 0 and material porosity. The reduction of ash content in the optimum stage can be attributed to the volatilization of organic constituents, thereby reducing the ash at high temperatures; however, acceptable ash content rate in commercial AC ranges from 2% - 10%. Also, the lower the volatile content, the higher the porosity of the adsorbent.

**Table 2:** Characterization of Optimum and minimum activated carbon.

Characteristics	Value of optimum activated carbon	Value of minimum activated carbon
Moisture Content (MC) (%)	4	5.6
Ash Content (%)	2.5	3
Volatile Matter Content (%)	30.2	20.8
Iodine Value (mg/gm)	1628.95	470.41

### 3.6 Characterization of sewage water

Table 3 observed that the BOD of the sewage water was high due to the presence of organic matter such as food wastes and faeces, among others. The higher the organic matter, the higher the number of bacteria present in the wastewater. Also, the sewage water is alkaline due to the decomposition of the organic matter while the TDS and TSS is considerably high.

**Table 3:** Characterization of sewage water.

Water Quality Parameter	Value
Biochemical Oxygen Demand (BOD) (mg/l)	288
pH	7.7
Total Dissolved Solids (TDS) (mg/l)	183.7
Total Suspended Solids (TSS) (mg/l)	232

### 3.7 Characterization of treated water with optimum and minimum activated carbon

The result of water treatment with optimum AC as presented in Table 4 shows a reduction in pH from alkaline close to the neutral point. Also, the BOD, TDS, and TSS were greatly reduced at the optimum condition due to the larger pore structure, which increased its capability to absorb molecules. However, the optimum AC is preferred to minimum AC due to its high operating conditions, which led to complete activation owing to higher activation time and temperature and larger pores developed due to higher IT.

**Table 4:** Characterization of water treated with Optimum and minimum activated carbon.

Characteristics	Value of optimum activated carbon	Value of minimum activated carbon
Biochemical Oxygen Demand (mg/l)	20	112.2
pH	7.12	7.6
Total Dissolved Solids (mg/l)	16.4	103
Total Suspended Solids (mg/l)	15.7	174

## 4 Conclusion

The optimum AC production from sawdust was found to be achieved in the optimum conditions of 1 mol phosphoric acid, 600 °C of carbonization temperature at 75 min time, and 30 h of impregnation time (IT). Also, the adsorption rate increases with IT up to 600 °C. The optimum AC produced is a suitable condition for the sewage water treatment due to a reduction in BOD, pH, TSS and TDS. However, AC's moisture content should be tested immediately after the carbonization stage to avoid an increase of MC due to a humid environment. Hence, further characterization should be done on sewage water to ascertain water fitness.

## Declarations

### Author contribution statement

Oladimeji Temitayo, E.: Conceived and designed the experiments; Analyzed and interpreted the data; Contributed reagents, materials, analysis tools or data; Wrote the paper.

Odunoye Babatunde, O.: Performed the experiments; Analyzed and interpreted the data; Contributed reagents, materials, analysis tools or data.

Elehinafe Francis, B., Obanla Oyinlola, R. & Odunlami Olayemi, A.: Conceived and designed the experiments; Wrote the paper.

### Funding statement

This research did not receive any specific grant from funding agencies in the public, commercial, or not-for-profit sectors.

### Data availability statement

Data will be made available on request.

### Declaration of interests statement

The authors declare no conflict of interest.

### Additional information

No additional information is available for this paper.
